# Development and prospective validation of COVID-19 chest X-ray screening model for patients attending emergency departments

**DOI:** 10.1038/s41598-021-99986-3

**Published:** 2021-10-14

**Authors:** Ignat Drozdov, Benjamin Szubert, Elaina Reda, Peter Makary, Daniel Forbes, Sau Lee Chang, Abinaya Ezhil, Srikanth Puttagunta, Mark Hall, Chris Carlin, David J. Lowe

**Affiliations:** 1 Bering Limited, London, UK; 2 NHS Greater Glasgow and Clyde, Glasgow, UK

**Keywords:** Diagnostic markers, Risk factors, Respiratory signs and symptoms, Respiratory tract diseases, Radiography

## Abstract

Chest X-rays (CXRs) are the first-line investigation in patients presenting to emergency departments (EDs) with dyspnoea and are a valuable adjunct to clinical management of COVID-19 associated lung disease. Artificial intelligence (AI) has the potential to facilitate rapid triage of CXRs for further patient testing and/or isolation. In this work we develop an AI algorithm, CovIx, to differentiate normal, abnormal, non-COVID-19 pneumonia, and COVID-19 CXRs using a multicentre cohort of 293,143 CXRs. The algorithm is prospectively validated in 3289 CXRs acquired from patients presenting to ED with symptoms of COVID-19 across four sites in NHS Greater Glasgow and Clyde. CovIx achieves area under receiver operating characteristic curve for COVID-19 of 0.86, with sensitivity and F1-score up to 0.83 and 0.71 respectively, and performs on-par with four board-certified radiologists. AI-based algorithms can identify CXRs with COVID-19 associated pneumonia, as well as distinguish non-COVID pneumonias in symptomatic patients presenting to ED. Pre-trained models and inference scripts are freely available at https://github.com/beringresearch/bravecx-covid.

## Introduction

An outbreak of severe acute respiratory syndrome coronavirus 2 (SARS-CoV-2) led to the COVID-19 pandemic of 2020^1^. The early clinical course of COVID-19, which often includes non-specific symptoms such as fever, dry cough, and dyspnoea, can be challenging for clinicians to distinguish from other respiratory illnesses^2–4^. Whilst most COVID-19 patients have a mild clinical course, a proportion of patients demonstrate rapid deterioration from the onset of symptoms into severe illness with or without acute respiratory distress syndrome (ARDS)^5,6^.

Effective screening of infected individuals is a critical step in the COVID-19 triage process^7,8^. Laboratory based real-time (RT) polymerase chain reaction (PCR) tests of nasopharyngeal swabs are considered as the gold standard for identifying clinical cases of infection. However, RT-PCR has several limitations^3,9^, including limited sensitivity (83.3%)^9,10^, a long turnaround time of up to 72 h, and requirements for specialist laboratory infrastructure and expertise^11^. Furthermore, some patients, including those with high clinical suspicion of COVID-19, test falsely negative on initial RT-PCR test, sometimes requiring multiple subsequent tests to return an eventual positive result. Antigen or molecular point-of-care tests offer rapid turnaround but with a drop in sensitivity for symptomatic patients (72%)^12^.

Thoracic imaging forms part of the COVID-19 assessment^13^ and plays an important role in early COVID-19 diagnosis^14^. In mainland China, CT was often the investigation of choice for COVID-19^14,15^. However, such practice was burdensome on radiology departments and challenging for infection control^16^. Most patients with dyspnoea undergo chest radiography (CXR) at presentation to hospital, with CXRs seen as first-line investigation of the COVID-19 pathway^17^. Indeed, while awaiting the RT-PCR result, most suspected COVID-19 patients are clinically diagnosed with the triad of clinical assessment, CXR, and blood tests.

Despite their utility, radiological interpretation of CXRs in suspected COVID-19 patients remains challenging due to the idiosyncratic nature of this disease. For example, no single feature on chest radiography is diagnostic of COVID-19 pneumonia^18^ and early or mild disease is often accompanied by a paucity of radiological signs^15,18^. Computer-aided diagnostic systems that can aid radiologists to more rapidly and accurately detect COVID-19 cases have been suggested as important operational adjuncts with potential to alleviate radiology workloads and improve patient safety^19^.

Several deep learning-based techniques have been introduced to identify COVID-19 pneumonia on frontal CXRs^20–24^. COVID-Net was one of the first neural network models tailored for COVID-19 diagnosis and released as an open-source framework^20^. More recently, DeepCOVID-XR, an ensemble of convolutional neural networks trained on a large multi-centre cohort of n = 14,788 images (n = 4253 COVID-19 positive) and validated on an external testing set from a single institution, performed on par with a consensus of five thoracic radiologists^24^.

Despite their rapid proliferation, AI models have been limited by either methodological weaknesses and/or underlying biases^25^. First, publicly-available images used to train COVID-19 deep learning models are often of variable quality and questionable validity^24,26^. Given the subtlety of radiological signs and challenges in their interpretation, high-resolution and multi-centre radiographs are needed to establish effective baselines. Second, neural networks have demonstrated propensity to learn features that are specific of the dataset more than the ones that are specific of the disease^27^, resulting in overestimated performance with poor generalisability potential^28,29^. This is exacerbated by increasing prevalence of “Frankenstein” datasets, that is, datasets assembled from multiple sources and redistributed under a new name^25^, leading to problems with algorithms being trained and tested on identical or overlapping datasets while believing them to be from distinct sources. Finally, training and testing set selection has often been carried out retrospectively with an equal balance between positive and negative cases as well as clear symptomatic differences between cases and controls. Given the rapidly changing prevalence of COVID-19 in the community and machine learning model sensitivity to class imbalance^30^, generalisability of COVID-19 classifiers to symptomatic patients with a clinical suspicion of COVID-19 infection remains poorly understood.

In this paper we analyse n = 293,143 CXRs (n = 1650 COVID-19 positive) across 14 acute sites in NHS Greater Glasgow and Clyde (GG&C) between March and May, 2020. We apply a patch-wise neural network training approach that takes advantage of high-resolution CXR imaging and evaluate prospective model performance on continuously collected CXRs (n = 3289, n = 249 COVID-19 positive) of patients presenting to EDs with COVID-19 symptoms across NHS GG&C in June–September, 2020. Finally, we compare the performance of our AI ensemble (CovIx) with interpretations of board-certified radiologists.

## Materials and methods

Delegated research ethics approval for this study (reference: 104,690/WP11/S1) was granted by the Local Privacy and Advisory Committee at NHS Greater Glasgow and Clyde. Cohorts and de-identified linked data were prepared by the West of Scotland Safe Haven at NHS Greater Glasgow and Clyde. In Scotland, patient consent is not required where routinely collected patient data is used for research purposes through an approved Safe Haven. This is set out by the Scottish Government in the Safe Haven Charter^31^. For that reason, informed consent is not required and was not sought. All research was performed in accordance with relevant guidelines/regulations.

### Dataset

**Figure 1:**
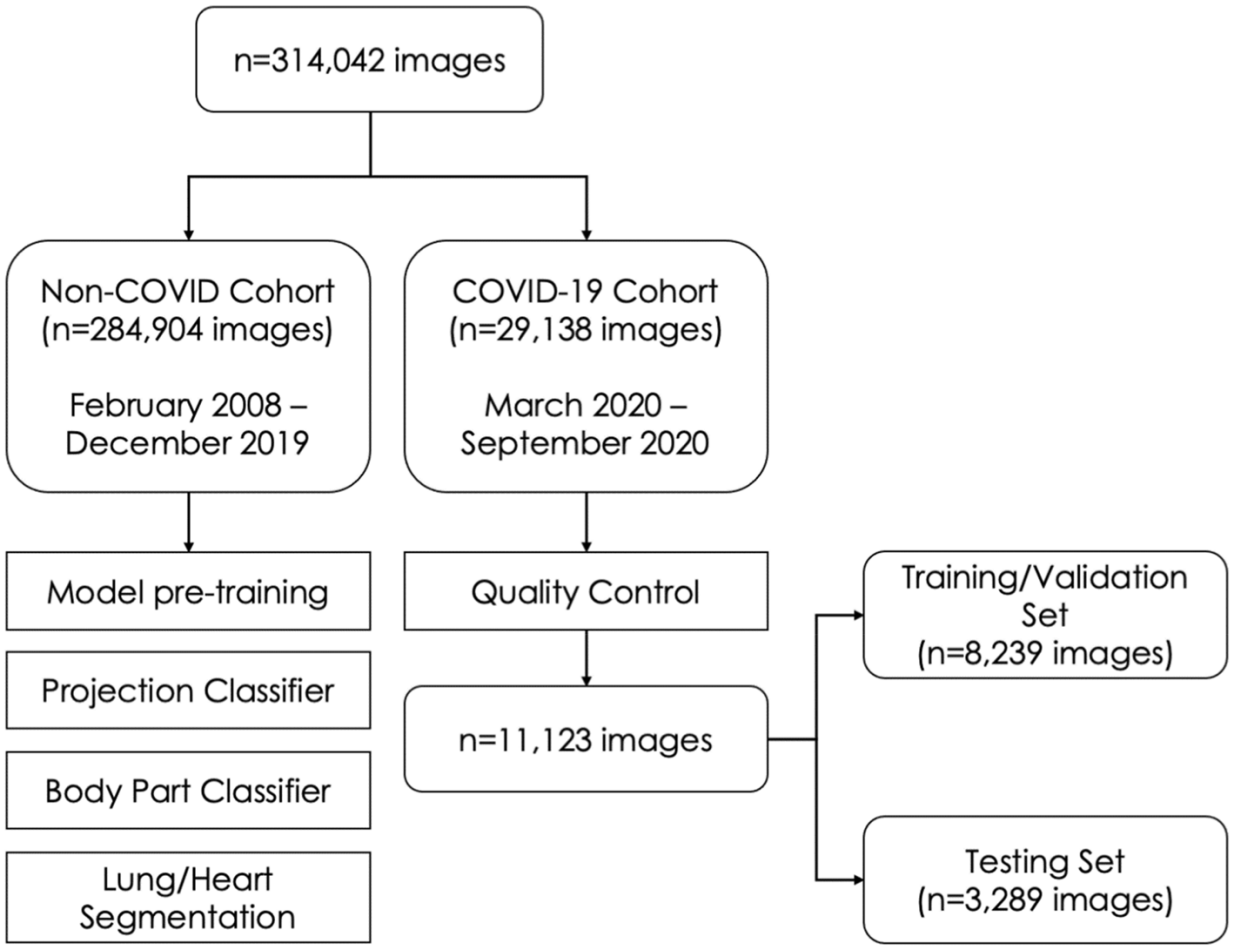
Dataset Characteristics. Entire dataset comprised of Non-COVID (n = 284,904) and COVID-19 (n = 29,138) cohorts. Images in the non-COVID cohort (collected between February 2008 and December 2019) were used to pre-train all classifiers and generate Projection and Body Part classifiers, as well as the Lung and Heart Segmentation model. Images in the COVID-19 cohort (collected between March 2020 and September 2020) have undergone rigorous Quality Control process and were used to train and test the COVID-19 Ensemble.

All chest radiographs in our dataset (Fig. 1, n = 314,042) were obtained between February 2008 and September 2020 across 14 acute sites in NHS GG&C.

**Figure 2:**
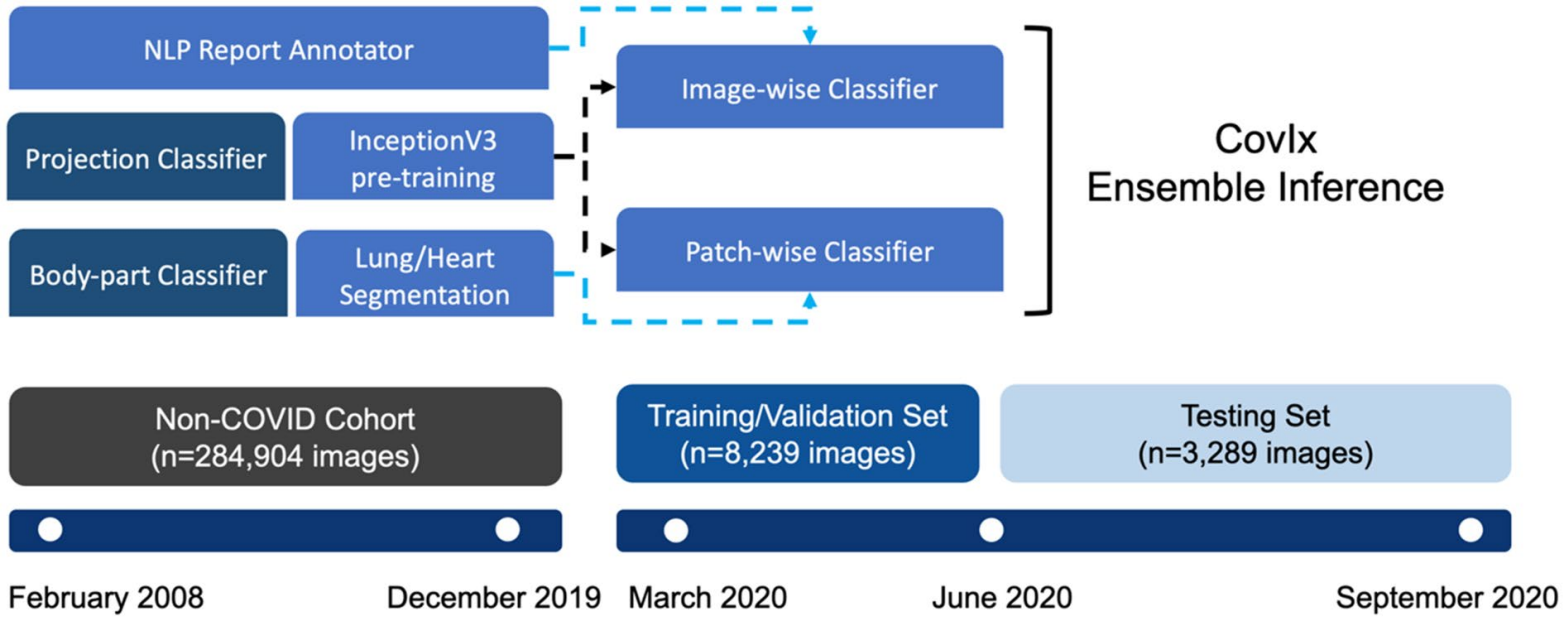
Study design used for the development and prospective validation of the CovIx ensemble. Non-COVID Cohort (n = 284,904 CXRs collected between February 2008 and December 2019) was used to pre-train all classifiers used in this work. CXRs collected between March and May 2020 were used to train CovIx model ensemble, whilst prospective evaluation was carried out on n = 3289 CXRs between June and September 2020.

Images were produced by 11 different X-ray systems, including those used for portable studies. The Non-COVID-19 cohort (n = 284,904 images) comprised of images collected prior to January 2020, whilst the COVID-19 cohort (n = 29,138 images) comprised of patients who were placed on the COVID-19 clinical pathway between March and September, 2020 (Fig. 2). Image resolution ranged from 253 × 902 to 4280 × 3520 pixels, with each pixel represented in grayscale with 16-bit precision. Identifiable patient data was removed from DICOM files and corresponding radiological reports using Named Entity Recognition algorithms within the Canon Safe Haven AI Platform (SHAIP). SHAIP is a trusted research environment constructed specifically for machine learning within the health board network and deployed in NHS GG&C through Industrial Centre for Artificial Intelligence Research in Digital Diagnostics (iCAIRD).

### Quality control and inclusion criteria

Prior to analysis, all images have undergone rigorous quality control procedure. First, images with width less than 1,500 pixels were excluded from the study. Second, DICOM Body Part Examined (0018, 0015) and View Position (0018, 5101) attributes were filtered by “Chest” and Anteroposterior (“AP”) or Posteroanterior (“PA”) respectively. In cases where attributes contained missing information, results were inferred using pre-trained deep neural network classifiers, retaining images with very high probability of Chest (> 0.99) and AP/PA (> 0.99) labels (see “Quality control classifiers”. Paediatric (patients < 16 years old) and follow up X-rays were excluded from this study.

### Ground truth generation and natural language processing

CXRs in the COVID-19 cohort were assigned one of the following labels—Normal, Abnormal Non-Pneumonia (“Abnormal”), Non-COVID-19 Pneumonia (“Pneumonia”), or COVID-19 (“COVID+”). Normal and Abnormal Non-Pneumonia classes were assigned to CXRs at the time of their interpretation by a reporting radiologist, whilst Non-COVID-19 Pneumonia label was assigned to CXRs with confirmed viral or bacterial pneumonia laboratory result during the associated clinical encounter.

COVID-19 positivity was assigned to a CXR if either (1) any single RT-PCR result was positive for SARS-CoV-2 within 14 days of image acquisition or (2) a diagnosis of COVID-19 by ICD-10 code during the associated clinical encounter. Patients with only documented negative RT-PCR tests for COVID-19 during their clinical encounter were labelled as COVID-19 negative.

Annotation of the free-text radiological reports was automated by training a custom DistilBERT Natural Language Processing (NLP) model^32^. The model was used to detect both normality of a radiological report^33^ and assign each report with one or more labels, including Atelectasis, Consolidation, and Effusion. An uncased DistilBERT model was initialised using weights provided by Sanh et al.^32^ We then continued to pre-train the model for three epochs using n = 2,067,531 full text PubMed articles distributed under Creative Commons (CC) BY or CC0 license^34^, totalling n = 224,427,218 sentences. All words were converted to lower case and punctuation was removed. Tokenization was performed using a custom WordPiece^35^ tokenizer with a vocabulary size of 52,000 words and word occurrence frequency of greater or equal to two. Finally, the pre-trained DistilBERT model was further finetuned using 1500 manually annotated free-text radiological reports (sourced from the non-COVID-19 cohort), with a batch size of four, for five epochs using Adam optimizer^36^ with a learning rate of 1 × 10^–5^ and Binary Cross-Entropy loss with logits. The finetuned multi-label DistilBERT model was trained to output probabilities of the following labels–Atelectasis, Pleural Calcification, Cardiomegaly, Consolidation, Effusion, Emphysema, External Medical Device, Fracture, Internal Medical Device, Interstitial Opacity, Metalwork, Nodule, Pleural Thickening, Other Abnormality, and No Findings. The labels were selected due to occurrence in at least 20 radiological reports from the training set. Model performance was validated on an independent dataset of n = 500 manually-labelled reports.

### Deep neural networks

#### Quality control classifiers

Two quality control (QC) classifiers were trained to differentiate (1) chest versus non-chest body part (“body part classifier”) and (2) AP versus PA projection (“projection classifier”). The Non-COVID-19 cohort (n = 284,904 images, non-COVID-19 cohort) was selected for both classifiers. Images were randomised into training (80%), validation (10%), and testing (10%) sets using stratified splits. To avoid data leakage, we ensured that patient identifiers do not overlap between splits.

QC classifiers were built using the InceptionV3^37^ architecture and initialised with ImageNet weights^38^. Global Average Pooling and two dense layers comprised the classification head. Softmax activation was applied to the final dense layer. Models were trained on 16-bit DICOM files with 32 images per batch using Adam optimizer with a learning rate of 1 × 10^–3^, whilst minimising the Categorical Cross-Entropy loss. Input images were resized to 299 × 299 using bilinear interpolation without preserving the aspect ratio. During training, images were subject to random augmentations, which included brightness adjustments, angular rotation, and left–right flipping. Training was terminated early if validation loss did not improve after ten consecutive epochs.

#### Ensemble of deep neural networks for COVID-19 prediction

All networks used in the CovIx ensemble utilise an InceptionV3 backbone and a classification head comprising of a Global Average Pooling layer, Dense layer (n = 1024 neurons), Dropout (dropout rate of 0.2) layer, and a final Classification layer (a Dense layer with number of neurons reflecting the number of desired classes). The InceptionV3 backbone produced the best performing-classifiers compared to VGG16, DenseNet, and ResNet both in our experiments as well as external studies^39^. Network weights for all InceptionV3 backbones were obtained by training a multi-label classifier to identify one or more of the NLP labels extracted from free-text radiological reports in n = 284,904 images from the non-COVID-19 cohort. (see Ground Truth Generation and Natural Language Processing).

**Figure 3:**
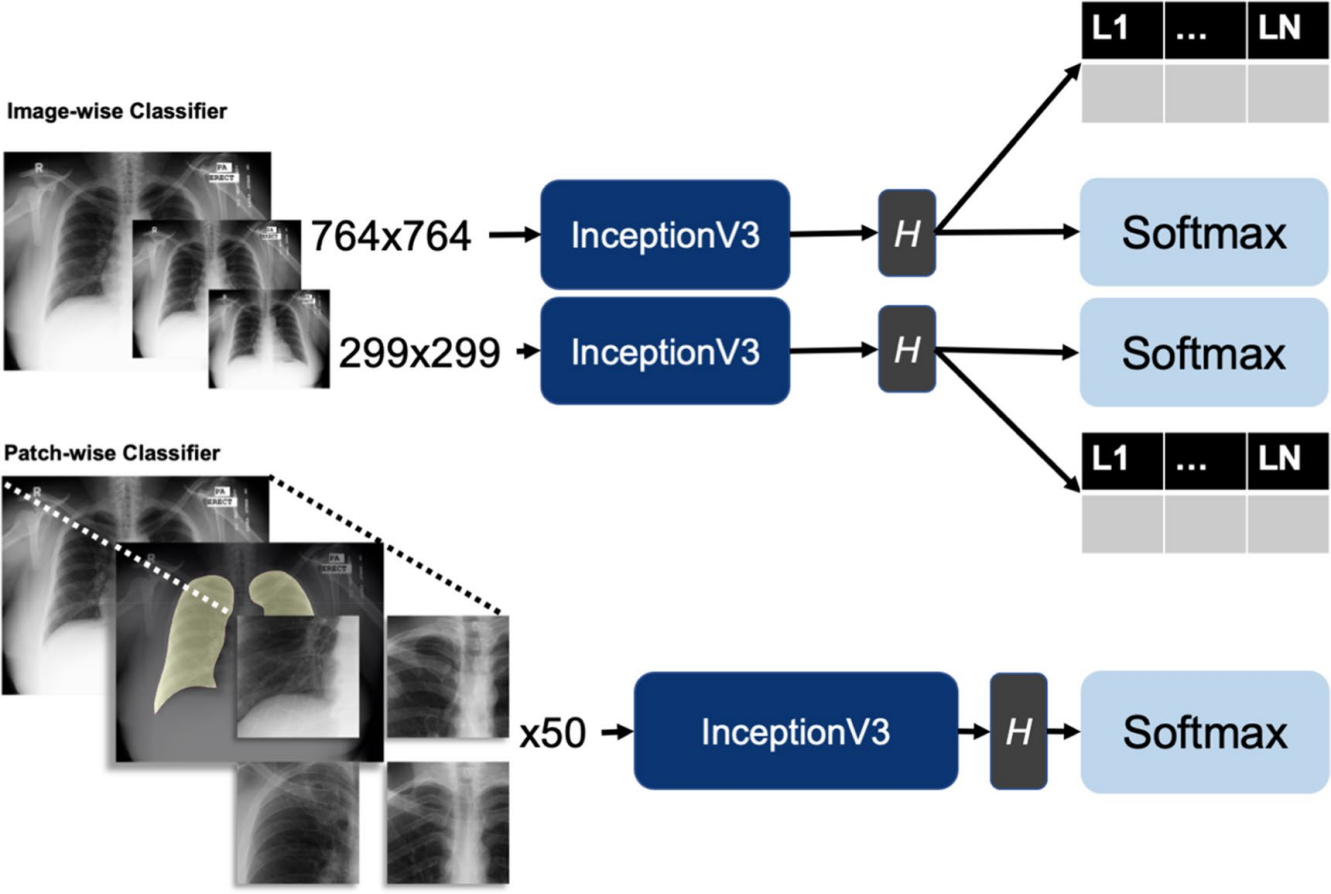
Constituents of the CovIx ensemble. The low- and high-resolution image-wise classifiers were trained on frontal CXRs scaled to 299 × 299 and 764 × 764 pixels respectively. The classification head (H) contained two outputs —an NLP multi-label classifier output (L*1*-L*N*) and a COVID-19 classifier (Softmax). The NLP output consisted of a Dense layer with a neuron per NLP target class (classes = 10) followed by a Sigmoid activation function, while the COVID-19 classifier output likewise consisted of a Dense layer with four output neurons representing Normal, Abnormal, Pneumonia and COVID + respectively followed by a Softmax output. The patch-wise classifier was built by scaling each image to 1500 × 1500 resolution, extracting lung and heart masks, and taking 50 random patches cropped to image masks with a size of 299 × 299 as the network inputs. At inference stage, 50 random patches were acquired for each image and fed to the classifier to generate class probability values for Normal, Abnormal, Pneumonia, and COVID + classes.

CovIx is an ensemble of three models (Fig. 3) designed to capture micro- and macro-level features of the dataset—the high-resolution patch-wise classifier, low resolution image-wise classifier, and a high-resolution image-wise classifier. The final probability value produced by the ensemble is the weighted mean of the output probabilities produced by the Softmax output of each constituent model.

The low- and high-resolution image-wise classifiers were trained on frontal CXRs scaled to 299 × 299 and 764 × 764 pixels respectively. When constructing the InceptionV3 networks with varying input shapes, the number of channels in each layer of the network remained constant, with only the dimensions of the intermediate feature maps being affected. The final feature map output prior to Global Average Pooling had a dimension of 8 × 8 in the 299 × 299 model and a dimension of 22 × 22 in the 764 × 746 model. The classification head contained two outputs—an NLP multi-label classifier and a COVID-19 classifier. The NLP multi-label classifier was trained to identify one or more of the NLP labels extracted from free-text radiological reports, whilst the COVID-19 classifier assigned a probability value to Normal, Abnormal Non-Pneumonia, Non-COVID-19 Pneumonia, and COVID + classes. The network was trained end-to-end, such that the NLP label outputs were used as auxiliary targets for the COVID-19 classifier. This auxiliary training objective served to regularize the network training by encouraging the neural networks to extract a variety of useful features from all input images, whether the COVID class was present or not, making the networks more generalizable and more resilient.

The patch-wise classifier was built by scaling each image to 1500 × 1500 resolution (the lowest DICOM resolution in our training set) and taking 50 random patches with a size of 299 × 299 as the network inputs (default InceptionV3 input size). To ensure that random patches represent meaningful information, the centres of each patch were randomly selected from segmented lung areas^40^. Segmentation masks were obtained by training a UNet model^41^ with a ResNet-50^42^ backbone and ImageNet weights on a collection of 2,000 manually labelled lung and cardiac fields. At inference stage, 50 random patches were acquired for each image and fed to the classifier to generate class probability values for Normal, Abnormal, Pneumonia, and COVID + classes. The final prediction was taken as the average class probability across 50 patches.

All models were trained on 16-bit DICOM files with 64 images per batch using Adam optimizer. For the models with multiple outputs (low- and high- resolution image-wise classifiers), the final loss function was the sum of the categorical cross entropy loss applied to the Softmax output and the binary cross entropy loss of the output of the NLP layer. The 16-bit DICOM images were linearly rescaled to the range [− 1, 1] before being fed into the models. A learning rate of 1 × 10^–4^ was applied to the neural network backbone, whilst layers within the classification head were trained with a learning rate of 1 × 10^–5^ to minimise effects of the double descent phenomenon^43^. Images were subject to random train-time augmentations, which included brightness adjustments, angular rotation, and left–right flipping. Training was terminated early if validation loss did not improve after ten consecutive epochs.

### Comparison with COVID-Net, DeepCOVID-XR, and consensus radiologist interpretations

COVID-Net^20^ and DeepCOVID-XR^24^ models were used to establish testing set performance reference standard. Briefly, COVID-Net, trained and validated on n = 13,975 CXRs (n = 358 COVID + images), utilises a bespoke convolutional network architecture to differentiate Normal, COVID-19, and non-COVID-19 Pneumonia CXRs, whilst DeepCOVID-XR, trained and validated on n = 14,788 CXRs (n = 4253 COVID + images), is an ensemble of 24 neural networks that assigns each CXR a probability of displaying signs of COVID-19. Prior to inference, all images in the prospectively-collected testing set were converted to 8-bit PNG files, preserving original resolutions. Pre-trained model weights were obtained from respective GitHub repositories and class probabilities calculated using the author-supplied inference scripts.

One hundred images were selected from patients presenting to ED in NHS GG&C in June 2020. Images were acquired over a continuous time period, representing “real-world” incidence of COVID-19 presentation. Expert interpretations were independently provided by four radiologists with 6 months to 4 years (average 2.5 years) post Fellowship of the Royal College of Radiologists examination. Radiologists were blinded to any identifying patient information or clinical characteristics.

### Statistical analysis

The predictive performance of the NLP and AI systems was assessed by using the area under the receiver operating characteristic (AU ROC) and precision-recall (AU PR) curves and 95% Confidence Intervals (CIs) were produced using 2000 bootstrap samples. Sensitivity, positive predictive value (PPV), and F1-score (a measure of accuracy, reflecting the harmonic mean of PPV and sensitivity, where 1 represents perfect PPV and sensitivity) were determined. Interobserver agreement was measured using Cohen’s Kappa. Model sensitivity and specificity were compared using McNemar’s test^44^ and AU ROCs were compared using DeLong test^45^. A two-tailed *p* value of 0.05 was considered statistically significant.

## Results

### Cohort characteristics

All CXRs in our dataset (n = 314,042) were obtained between February 2008 and September 2020 across 14 acute sites in NHS GG&C. Of the 314,042 images, n = 2,313 (0.74%) and n = 253,141 (80%) had missing Body Part Examined (0018, 0015) and View Position (0018, 5101) DICOM attributes respectively. To extrapolate the missing attribute values, we trained two classifiers that determine whether an X-ray is a chest radiograph (body part classifier) and whether its projection is AP or PA (projection classifier). Both classifiers achieved AUROC > 0.99 on a held-out testing set and were used to inform our quality control procedure (see “Methods”).

Of the 29,138 images in the COVID-19 cohort, n = 11,123 images (38%) from 8,511 patients passed our inclusion and QC criteria (4407 females, average age of 66, range 16–105 years, see “Methods”). The training set consisted of n = 8239 images obtained from patients presenting across 14 acute sites in NHS GG&C with symptoms of COVID-19 between March and May, 2020. Of these 63% (n = 5190) were obtained in ED, whilst remaining were obtained in in-patient facilities. The testing set images were collected continuously in ED from symptomatic NHS GG&C patients presenting between June and September 2020 (Table 1). The rate of positivity for COVID-19 among chest radiographs in the test set (249/3,289; 7.6%) was lower than in the training (1,650/8,239; 20%). The proportion of anteroposterior radiograms was congruent between training and testing sets (28%).

**Table 1:** Patient characteristics in the training and testing sets.

	Size (number of images)	Age* (years)	Female Sex (number of images)	Anteroposterior frequency	COVID-19 positive (number of images)
Training Set	8239	67 ± 18	4090	28%	1650
Testing Set	3289	64 ± 18	1664	28%	249

*Reported as average ± standard deviation.

### Construction of CovIx ensemble for COVID-19 diagnosis

CovIx is a neural network ensemble that aims to capture macro- and micro-level features of the disease. All ensemble constituents utilise an InceptionV3 backbone, pretrained on CXRs from the non-COVID-19 cohort (n = 284,904 images). The pre-training task was a multi-label classification problem that aimed to assign a CXR with one or more of the 15 labels—Atelectasis, Pleural Calcification, Cardiomegaly, Consolidation, Effusion, Emphysema, External Medical Device, Fracture, Internal Medical Device, Interstitial Opacity, Metalwork, Nodule, Pleural Thickening, Other Abnormality, and No Findings.

To automate label extraction from free-text radiological reports, we trained a bespoke DistilBERT model using n = 2,067,531 full text PubMed articles (see “Methods”). NLP model performance on an independent set of 500 reports across the 15 labels achieved micro-average AUROC of 0.94 (AUROC_External Medical Device_ = 0.71 to AUROC_Abnormal Other_ = 1.0). NLP labels were subsequently assigned to all CXRs. Following multilabel pre-training, weights of the InceptionV3 model were transferred for further finetuning on the COVID-19 cohort.

CovIx ensemble is comprised of four components (Fig. 3) – (1) lung segmentation network, (2) high resolution patch-wise classification network, (3) low resolution image-wise classifier, and (4) high resolution image-wise classifier. The lung segmentation model was trained and validated on n = 2000 manually labelled lung fields. The resulting masks were used to select centres of the 50 random patches for every CXR, ensuring that only relevant information is captured. We have systematically assessed patch-wise model AUROC on a validation set using 10, 25, 50, and 100 patches per image. AUROC metric increased proportionally to the number of patches, with 50 and 100 patches producing identical validation set performance. The final patch-wise model consisted of 50 random patches, representing a balance between required computational resources and model performance.

Low- and high-resolution networks utilised 299 × 299 and 764 × 764 sized images respectively. The networks were trained to label each image with one or more of the 15 NLP labels extracted from free-text reports and subsequently use label probabilities to classify an image as Normal, Abnormal, Pneumonia, or COVID+. Final CovIx class probabilities were obtained by averaging outputs produced by constituent classifiers.

### Model performance

**Figure 4:**
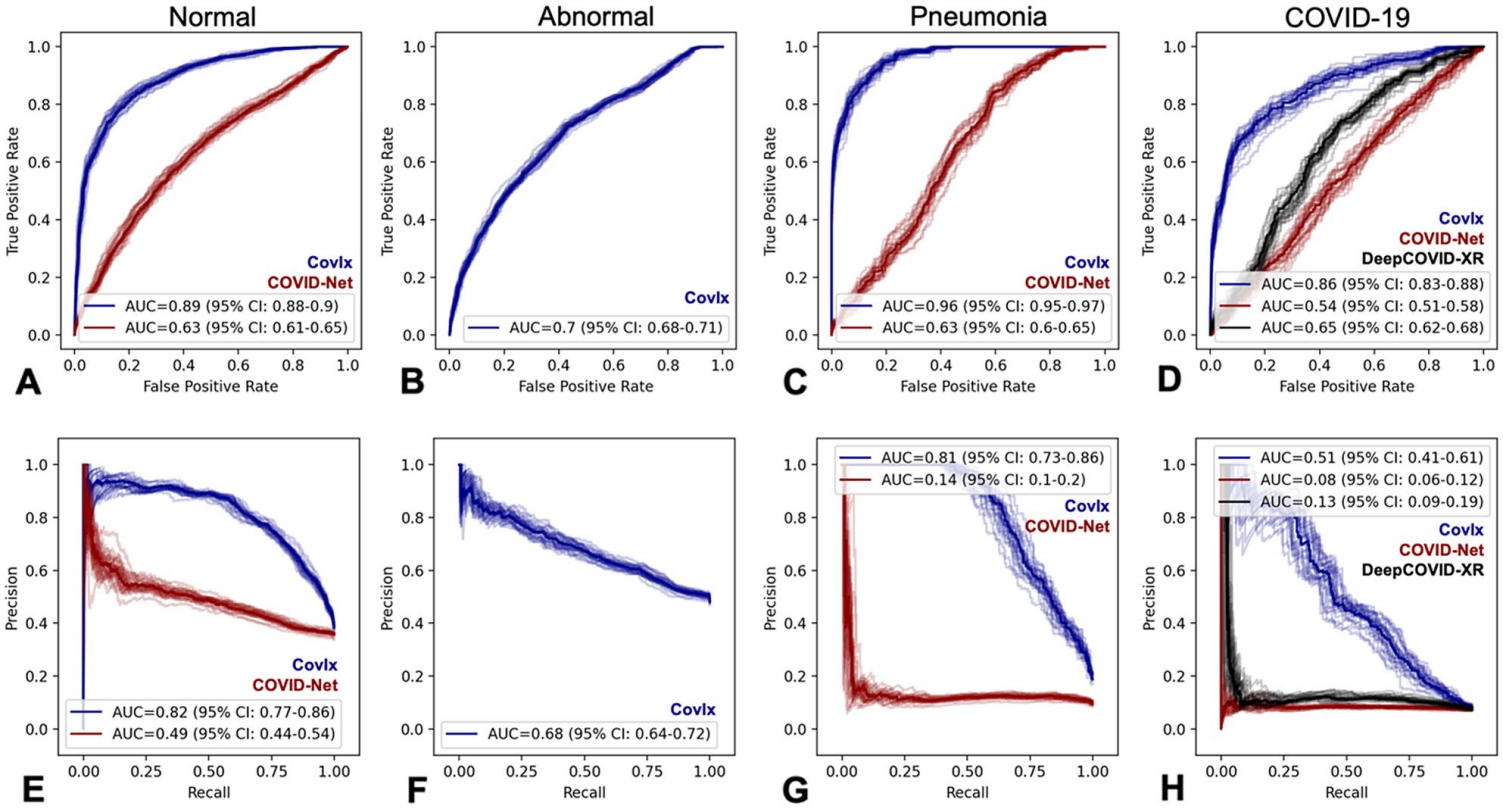
Discriminative performance of Chest-Xray classification algorithms on prospectively collected testing set of 3289 images. Discriminative capacity of CovIx (blue), COVID-Net (red), and DeepCOVID-XR (black) is represented as Receiver Operating Characteristics (ROC) (**A-D**) or Precision-Recall (PR) (**E-H**) curves. 95% Confidence Intervals (CI), generated using 2000 bootstrap samples, are visualised as pale curves.

CovIx performance was evaluated on a prospective continuously-collected testing set of n = 3289 images (n = 249 COVID-19 positive, collected June – September 2020) obtained from patients referred to the COVID-19 pathway following ED presentation in NHS GG&C. Performance of individual CovIx models is shown in Supplementary Fig. S1. The CovIx ensemble identified COVID-19 CXRs with AUROC and AUPR of 0.86 and 0.51 respectively (sensitivity = 0.55, PPV = 0.40, and F1-score = 0.47 [Fig. 4, Table 2]). Concurrent model identification of Normal, Abnormal, and Pneumonia CXRs resulted in AUROCs of 0.89, 0.70, and 0.96 respectively (Fig. 4).

**Table 2:** Model performance comparison in identifying COVID-19 CXRs.

Model	Label	Sensitivity	PPV	F1-score	AUROC	AUPR
CovIx	COVID-19	0.55	**0.40**	**0.47**	**0.86**	**0.51**
COVID-Net	COVID-19	**0.99**	0.07	0.14	0.54	0.08
DeepCOVID-XR	COVID-19	0.07	0.14	0.09	0.65	0.13

Top results are shown in bold.

**Figure 5:**
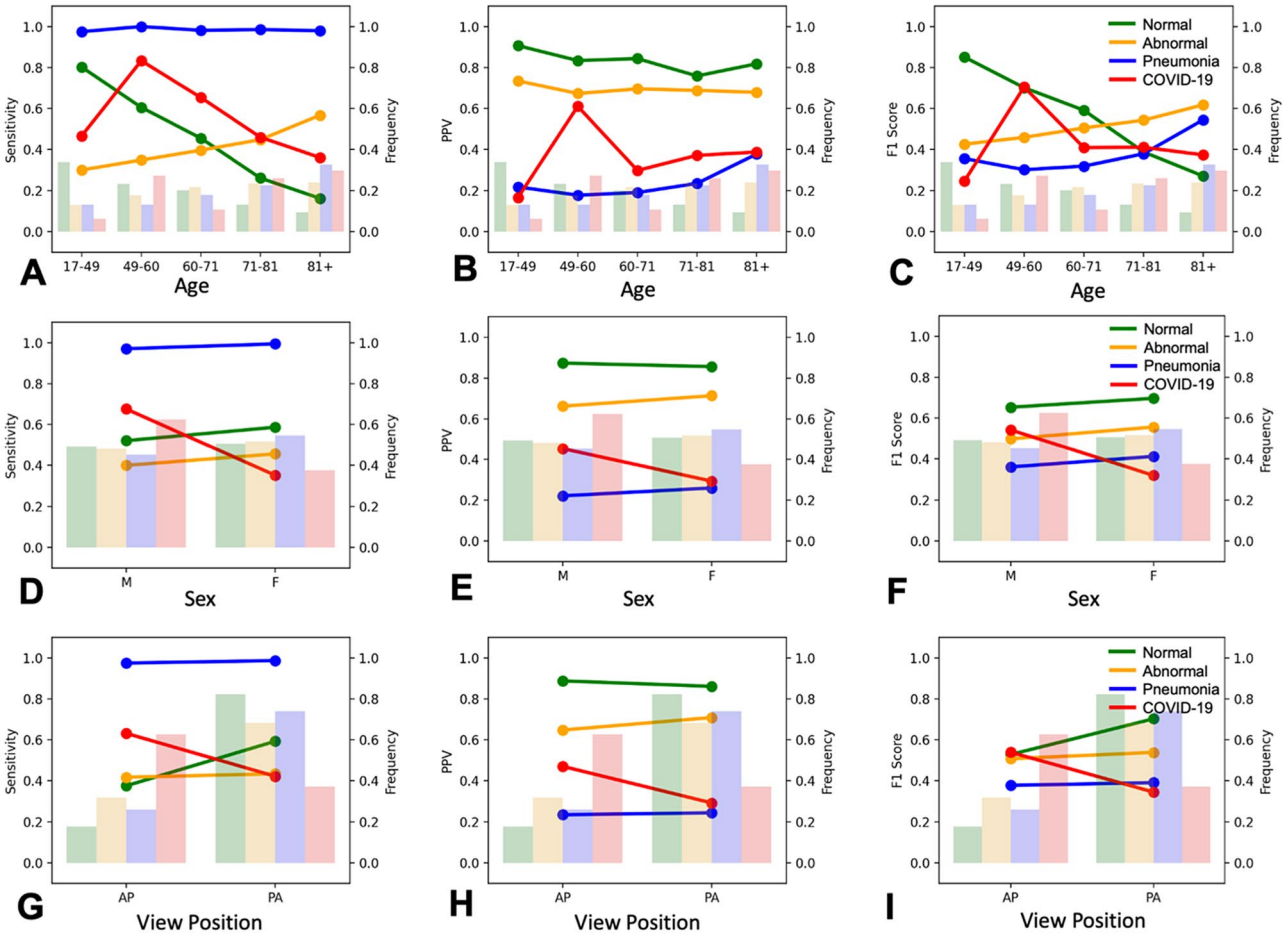
Effect of patient age, sex, and view position on CovIx performance. Model performance, expressed as sensitivity, positive predictive value (PPV), and F1 Score is represented as lines, whilst class frequencies, expressed as proportion of total images in the testing set, are shown as bars.

Impact of age on model performance was assessed by evaluating sensitivity, PPV, and F1-scores for every age quintile. The model achieved peak COVID-19 sensitivity (0.83), PPV (0.61), and F1-score (0.71) in the 49–60 age group (2nd age quintile) (Fig. 5). Furthermore, CovIx demonstrated increased COVID-19 detection in AP views and Male patients, exemplified by increased sensitivities (0.63, 0.68), PPVs (0.47, 0.45), and F1-scores (0.54) (Fig. 5).

To determine whether CovIx identifies COVID-19-specific features from CXRs, we applied the algorithm to n = 5000 randomly selected radiographs (n = 2819 normal radiological reports) obtained from patients presenting to NHS GG&C ED between September 2009 and August 2019. CovIx labelled 156 images (3%) as having radiological signs indicative of COVID-19. Of the 156 images 80 (51%) had normal radiological reports, 15 (10%) exhibited basal consolidations, and two (1%) had laboratory-confirmed Pneumonia. Remainder exhibited a diverse range of radiological signs, including cardiomegaly, emphysema, and atelectasis.

Finally, we compared CovIx algorithm to state-of-the-art, by evaluating COVID-Net and DeepCOVID-XR algorithms on our continuously-collected testing set. CovIx achieved better performance, expressed through significantly greater (DeLong *p* < 0.05) AUROC and AUPR values compared to other algorithms (Fig. 3) as well as higher PPV and F1-scores (Table 2).

### Comparison with expert radiologists

**Figure 6:**
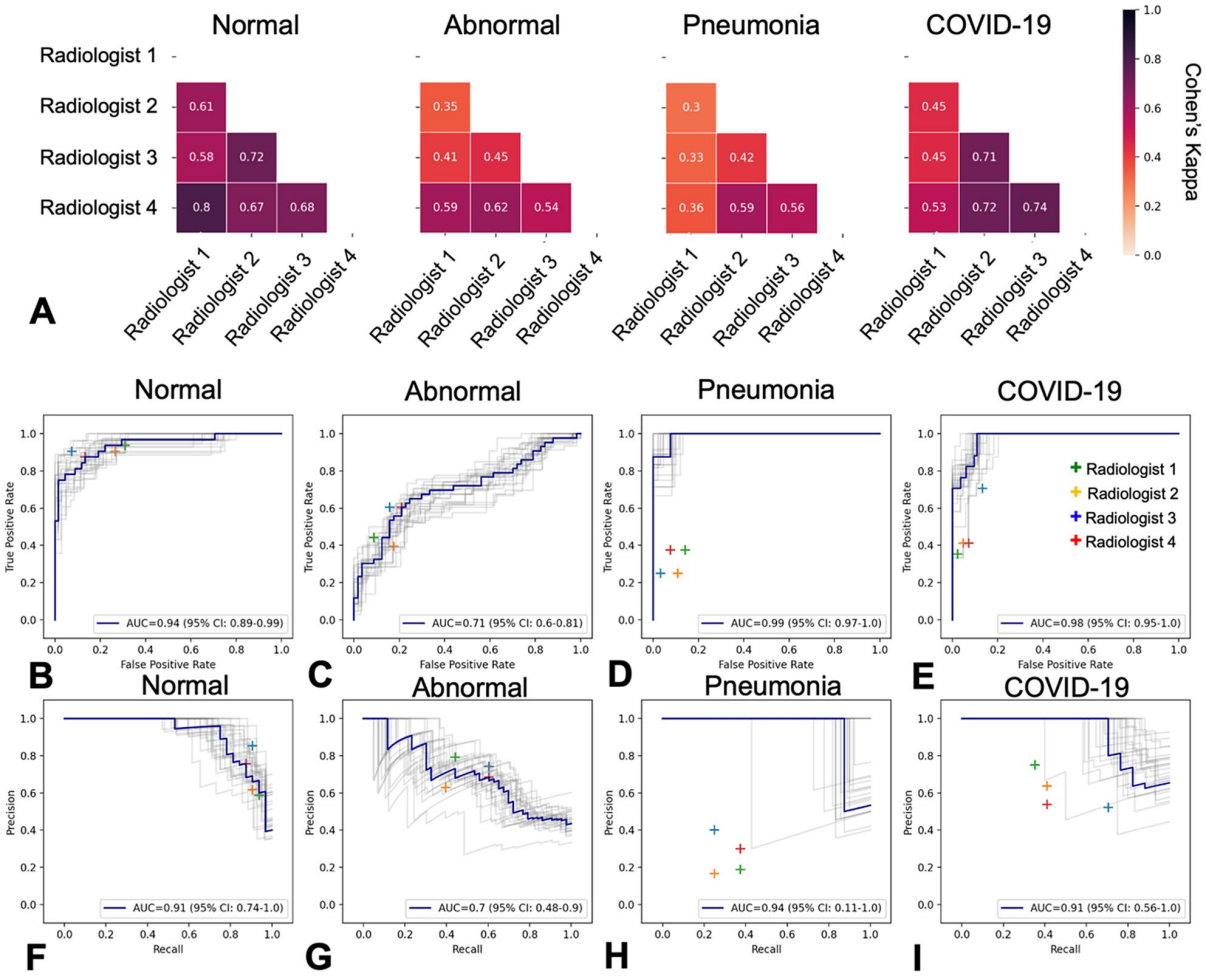
Comparison with board-certified radiologist interpretations. (**A**) Heatmaps visualising inter-reader agreement for Normal, Abnormal, Pneumonia, and COVID-19 images. (**B-E**) Receiver Operating Characteristic (ROC) curves and (**F-I**) Precision-Recall (PR) curves demonstrating CovIx model performance on the first 100 images collected in June 2020 across EDs in NHS GG&C (n = 17 COVID-19 positive). Individual radiologist performance is visualised as “ + ”. Grey lines indicate 95% Confidence Intervals (CI) calculated on 2000 bootstrap samples.

CovIx predictions were compared to board-certified radiologist interpretations on the first 100 continuously-collected CXRs of patients presenting to ED in June 2020 (n = 17 COVID-19 positive). Average inter-reader agreement, expressed as Cohen’s Kappa, for Normal, Abnormal, Pneumonia, and COVID-19 CXRs was 0.68, 0.49, 0.43, and 0.60 respectively (Fig. 6A).

The overall multi-class accuracy of CovIx on this test set was 60% compared with the reference standard, while the accuracy of individual radiologists ranged from 55 to 69% and the accuracy of the consensus interpretation of all four radiologists was 66%. Differences in overall performance were not statistically significant between CovIx and consensus radiologists’ labels (McNamara’s *p* value = 0.48, Supplementary Fig. S2).

At single-label level, CovIx performance was comparable to radiologists in Normal and Abnormal CXRs (McNamara’s *p* value = 0.82 and 0.53 respectively, Fig. 6B-D, F-H). However, CovIx exhibited statistically significant performance improvements in Pneumonia and COVID-19 classes (McNamara’s *p* value = 0.02 and 0.04 respectively, Fig. 6E,I), further exemplified through greater sensitivity, F1-score, and PPV (Table 3, Fig. S2).

**Table 3:** Comparison of model performance with Radiologist labels.

Class	Radiologist 1	Radiologist 2	Radiologist 3	Radiologist 4	Radiologist Consensus	CovIx
*Accuracy*						
Normal	**0.92**	0.79	0.77	0.87	0.88	0.86
Abnormal	**0.74**	0.64	0.71	0.71	0.72	0.67
Pneumonia	**0.91**	0.84	0.82	0.88	0.88	0.73
COVID-19	0.84	0.86	0.87	0.84	0.86	**0.94**
*Sensitivity*						
Normal	0.91	0.91	**0.94**	0.88	**0.94**	0.62
Abnormal	0.60	0.40	0.44	0.60	**0.63**	0.42
Pneumonia	0.25	0.25	0.38	0.38	0.25	**1.0**
COVID-19	0.71	0.41	0.35	0.41	0.41	**0.82**
*F1-score*						
Normal	**0.88**	0.73	0.72	0.81	0.83	0.74
Abnormal	**0.67**	0.49	0.57	0.64	0.66	0.52
Pneumonia	0.31	0.20	0.25	0.33	0.25	**0.37**
COVID-19	0.60	0.50	0.48	0.47	0.50	**0.82**
*PPV*						
Normal	0.85	0.62	0.59	0.76	0.75	**0.91**
Abnormal	**0.74**	0.63	0.79	0.68	0.69	0.69
Pneumonia	**0.40**	0.17	0.19	0.30	0.25	0.23
COVID-19	0.52	0.64	0.75	0.54	0.64	**0.82**

Top results are shown in bold.

## Discussion

In this paper we present development and prospective evaluation of an AI algorithm—CovIx—for screening of putative COVID-19 CXRs in symptomatic patients presenting to emergency department. The study population, aggregated across NHS GG&C, is representative of “real-world” patients presenting to ED between the peaks of the COVID-19 pandemic. On a continuously-collected testing set of n = 3,289 images (n = 249 COVID-19 positive), CovIx achieved AU ROC and AU PR of 0.86 and 0.51 respectively, outperforming state-of-the-art COVID-Net and DeepCOVID-XR models. Additionally, on a continuously-collected sample of 100 test images, CovIx performed favourably when compared to four board-certified radiologists, achieving statistically significant performance improvements for Pneumonia and COVID-19 identification.

Our work introduces several advantages. First, we use an ensemble approach that evaluates macro- and micro-level features of COVID-19 CXRs. The image-wise classifiers (macro-level) were pre-trained on n = 284,904 images using ground truths derived from a state-of-the-art NLP model trained on 224,427,218 sentences from medical literature. To the best of our knowledge this represents the largest medical corpus in a language modelling task^46^, providing high-quality annotations. Second, the patch-wise classifier (micro-level) enabled training on a relatively small training set, whilst still outperforming state-of-the-art models, such as DeepCOVID-XR. A similar approach, utilising 100 random patches during inference step, has been previously proposed^40^. We demonstrate that training a model using 50 random patches obtained from CXR lung fields, combined with a simple image augmentation schedule, yields superior performance. Third, AI models for COVID-19 detection have focused either on a binary COVID versus non-COVID classification task^24,47^ or on differentiation of COVID-19 pneumonia from viral or bacterial pneumonias^20,21,48^. Our algorithm introduces simultaneous detection of normality, COVID-19 pneumonia, viral or bacterial pneumonias, as well as non-pneumonia abnormalities. This approach makes it more versatile in diverse clinical environments such as the ED, where earlier diagnosis of bacterial pneumonia reduces mortality and length of stay^49^. Finally, most AI studies have been carried out at the time of considerable load on the healthcare system, with over-represented prevalence of COVID-19. As such, it is unclear how well these algorithms perform when COVID-19 is not the dominant viral pneumonia.

In this work, we rely heavily on the InceptionV3 architecture, which produced better performance compared to VGG16, DenseNet, and ResNet both in our experiments as well as external studies^39^. However, deep neural network models may suffer from over-fitting when there is a small number of training exemplars^50^, whilst shallow architectures may achieve comparable results with shorter training times^51^. Shallow architectures have already been explored in the context of COVID-19 screening^52,53^ and may provide a plausible alternative in cases where limited training data is available.

We demonstrate first evidence of AI performance in “real-world” settings on continuously collected CXRs in patients presenting to ED between the peaks of the pandemic. As such, our experiments reflect the changing prevalence of COVID-19 in the symptomatic ED population (20% March–May, 2020 vs. 8% June–September 2020). The training and testing sets represent an imbalanced machine learning problem, whereby the prevalence of a positive class (COVID-19) is considerably lower than that of the negative class (Normal, Abnormal-Other, Non-COVID Pneumonia). When class imbalance exists, learners will typically over-classify the majority group due to its increased prior probability^54^. To address this phenomenon, both undersampling the majority class and over-sampling the minority class have been proposed^55,56^. Generating synthetic samples through linear interpolation between data samples belonging in the same minority class^57^ or weighing the training loss function^58^ have also been suggested. These techniques assume that the prevalence of the minority class is a known and stable quantity, however prevalence of SARS-CoV-2 is changing rapidly^59^. To mitigate the impact of class imbalance in our models, we pre-trained every constituent of the CovIx Ensemble using a large collection of frontal CXRs (n = 284,904) obtained from patients prior to emergence of COVID-19 (Non-COVID Cohort, Figs. 1, 2, see “Methods”). This approach has been demonstrated to improve model robustness against imbalance and shown to outperform techniques such as over-/under-sampling and Synthetic Minority Oversampling Technique (SMOTE)^60^.

Furthermore, evaluation of CovIx on 5000 CXRs collected between September 2009 and August 2019, where COVID-19 prevalence is expected to be 0%, the algorithm identified only 156 images with high likelihood of COVID-19, suggesting that the algorithm is highly specific (97%). We believe this sets realistic expectations of AI performance.

Errors made by our algorithm were explainable. Of the 226 images with negative RT-PCR findings classified as COVID-19 positive by CovIx (false positives), 196 (87%) demonstrated signs including co-occurrence of bilateral small pleural effusions and unilateral lower lobe consolidation. Although individually these findings are present in a minority of COVID-19 patients^61^, presence of multiple abnormalities on a single CXR resulted in greater COVID-19 probability values. Similarly, of the 105 images with positive RT-PCR findings classified as non-COVID-19 (false negatives), only 23 (22%) had typical COVID-19 findings, such as multifocal ground glass opacity, linear opacities, and consolidation.

Due to variabilities in COVID-19 severity across our testing cohort, it’s likely that false negative predictions reflect limitations of CXR imaging rather than the algorithm itself. For example, 56% of symptomatic COVID-19 patients can demonstrate normal chest imaging, especially early in their disease course^14,18^. Additionally, many of the findings seen in COVID-19 imaging are non-specific and overlap with other viral pneumonias^62^. Consequently, CXR imaging alone is not recommended for COVID-19 diagnosis, but should be used concomitantly with clinical assessment, blood tests, and RT-PCR^17^. As such, our model, either on its own or in consort with other biomarkers/clinical findings, could play an important triage role in earlier identification of patients likely to have COVID-19, enabling improved flow and infection control.

A number of research and industrial groups have published deep learning-based studies and non-peer reviewed preprints^20–24,40,48,63^. Although the studies report extremely high sensitivity and specificity of AI algorithms to detect COVID-19 on CXRs, most have been limited by small sample sizes or have relied on images from publicly available datasets of variable quality and label accuracy^64^. Although larger open access COVID-19 datasets are becoming more prevalent, for example the COVIDx dataset comprising of 13,975 CXR images across 13,870 patient cases^20^, the utility of these resources is uncertain. Indeed, aggregation of disease-specific CXR datasets to produce a meta-training set can often lead to overinflated performance metrics^25^. Given that neural networks have propensity to learn features that are specific of the dataset more than the ones that are specific of the disease^27^, resulting models generalise poorly to independent testing sets^28,29^. We demonstrate this characteristic by assessing performance of the COVID-Net model our testing set. The model classified 98% of all images as COVID+ , resulting in poor PPV, AU PR, and AU ROC values (Fig. 4).

Murphy et al.^47^ present an evaluation of a commercial patch-based convolutional neural network, CAD4COVID-Xray, on a cohort of continuously acquired CXRs (n = 454) obtained in patients suspected of having COVID-19 pneumonia presenting to a single centre between March 4 and April 6, 2020. The network was first trained on a large collection of CXRs for tuberculosis detection and subsequently finetuned using publicly-available pneumonia dataset (n = 22,184 images)^65^ and internally-curated COVID-19 images (n = 416). The AI system correctly classified chest radiographs as COVID-19 pneumonia with an area under the receiver operating characteristic curve of 0.81. By contrast, our system was trained on four times as many COVID-19 cases obtained across 14 different institutions. Furthermore, our testing set represents “real-world” incidence of COVID-19 positivity (249/2,889 images, 9%) among patients presenting with symptoms of COVID-19 to ED.

More recently, an ensemble of 24 neural networks, DeepCOVID-XR^24^, has demonstrated high accuracy of COVID-19 detection (AUROC = 0.90 compared to RT-PCR reference standard) and compared favourably to consensus of five thoracic radiologists (AUROC = 0.95) on an independent testing set. The network was pre-trained on a large CXR dataset of over 100,000 images^66^ and finetuned on 14,788 frontal CXRs (4,253 COVID-19 positive) from 20 sites, producing a binary prediction of COVID-19 likelihood. Evaluation of DeepCOVID-XR on our testing set demonstrated considerable performance boost compared to the COVID-Net model (AUROC = 0.65, AUPR = 0.13, Table 2). Nevertheless, DeepCOVID-XR did not perform on par with the CovIx ensemble (AUPR_DeepCOVID-XR_ = 0.13 vs. AUPR_CovIx_ = 0.51). Given similar inclusion criteria (RT-PCR positivity during a clinical encounter), and study population characteristics (comparable age and gender profiles), it’s likely that technical differences account for discrepancies in DeepCOVID-XR performance^67^. For example, DeepCOVID-XR training and testing sets contained more AP images (89% and 97% respectively), compared to only 28% in our study population. Patients undergoing AP examination are more likely to exhibit severe symptoms with increasingly discernible signs of COVID-19 infection^68^. This is further supported by improved CovIx performance on AP projections (Fig. 5G-I). Previous studies also report that AP CXRs have shown an overall better inter-rater agreement for COVID-19 diagnosis compared to PA^68^.

CovIx ensemble performed best in patients within the 49–60 age group (2^nd^ age quintile) (Fig. 5A-C). Young age has previously been associated with increased likelihood of false negative findings on CXR in retrospective multi-institutional study, of 254 RT-PCR verified COVID-19 positive patients^69^. Additionally, older patients are more likely to present with more severe symptoms and multiple lobe involvement than young and middle-age groups^70^.

Notably, whilst model performance for Normal, Abnormal, and Pneumonia classes was independent of patient sex, CovIx demonstrated decreased performance in female patients, as exemplified by reduction in sensitivity, F1-Score, and PPV (Fig. 5D-F). Sex differences in COVID-19 severity and outcomes are well documented^71–73^, with men exhibiting more severe symptomatology, increased likelihood of intubation, and greater chances of mortality. CT imaging has also demonstrated significantly greater severity scores in men with a trend toward more bilateral lung involvement^74^. Additionally, breast tissue may project onto lung fields, thus increasing the density of the lung periphery and simulating ground-glass opacities^75^. To the best of our knowledge this is the first report of sex-related accuracy differences in AI-guided COVID-19 diagnosis using CXR imaging.

Our study has several limitations. First, the inclusion criteria was broadened to ensure sufficient numbers of COVID-19 positive images in our training set. As high-quality COVID-19 CXRs become more readily available, it’s likely that model performance can be refined further by building bespoke classifiers for AP and PA projections as well as opportunities to address age- and sex-driven discrepancies in model performance. Second, the performance of our algorithm was compared to RT-PCR as a reference standard, which itself has limited sensitivity due to sampling error or viral mutation^76^. Third, although we used a continuously collected testing set for model validation, we did not assess model performance in an independent institution. Therefore, the generalisability potential of our algorithm is unclear. Finally, CovIx is limited to only a single data type – frontal CXRs. It is anticipated that inclusion of multimodal dataset in clinical decision support will further improve model accuracy, reliability, and interpretation^77^. To support this area of research, we made the pre-trained CovIx models and inference scripts available to the research community (https://github.com/beringresearch/bravecx-covid).

Overall, we present and evaluate a deep learning algorithm for detection of COVID-19 infection in symptomatic patients presenting to emergency department. The algorithm was trained on a large representative population and tested on continuously collected data in a “real-world” setting. CovIx has the potential to mitigate unnecessary exposure to COVID-19 in busy ED settings by serving as an automated tool to rapidly triage patients for further testing and/or isolation. Planned future studies include (1) incorporation of imaging data with readily-available point-of-care clinical data such as demographics and vital signs to further boost the performance, (2) evaluation of model generalisability in external institutions outside NHS GG&C, and (3) adoption of the algorithm for risk prediction of clinically meaningful outcomes in patients with confirmed COVID-19. By providing the CovIx code base as an open-source project, we hope investigators will further improve, fine-tune, and test the algorithm using clinical images from their own institutions.

## Supplementary Information

Supplementary Information.
