# Supplementary material for: Development and prospective validation of COVID-19 chest X-ray screening model for patients attending emergency departments
Source: Sci Rep. 2021 Oct 14;11:20384. doi: 10.1038/s41598-021-99986-3 (PMC8516957; doi:10.1038/s41598-021-99986-3)
Supplement: Supplementary file 1 — Supplementary Information. [file 41598_2021_99986_MOESM1_ESM.docx]

# Supplemental Information: Development and prospective validation of COVID-19 chest X-Ray screening model for patients attending emergency department

Ignat Drozdov^1*^, Benjamin Szubert^1^, Elaina Reda^2^, Peter Makary^2^, Daniel Forbes^2^, Sau Lee Chang^2^, Abinaya Ezhil^2^, Srikanth Puttagunta^2^, Mark Hall^2^, Chris Carlin^2^, David J Lowe^2^

1. Bering Limited, United Kingdom
2. NHS Greater Glasgow and Clyde

*address correspondence to: idrozdov@beringresearch.com

## Supplemental Figures

**
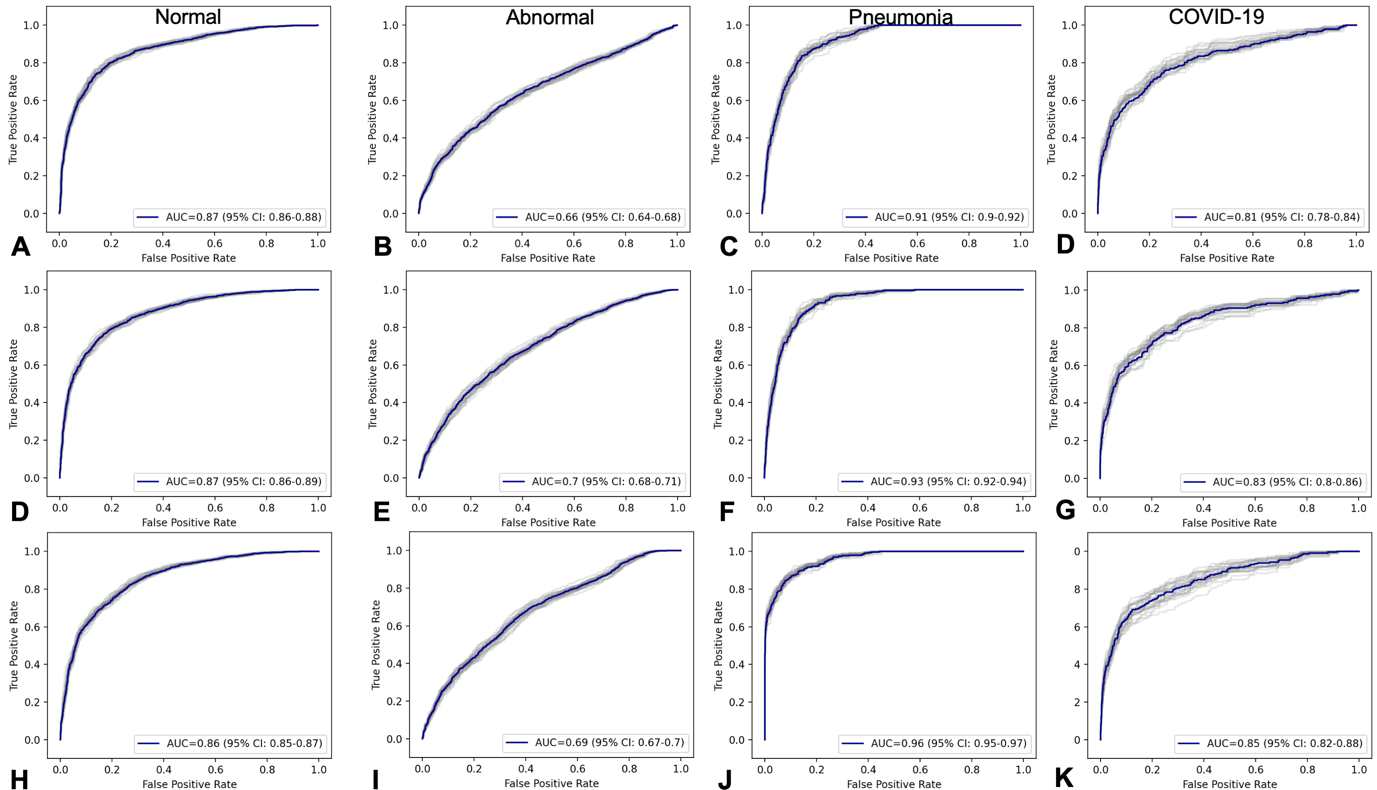
**

**Figure S1. Performance of individual constituents of the CovIx classifier.**

Receiver Operating Characteristic curves demonstrating discriminative capacity of constituent models of the CoviIx algorithm. Multi-outouput Inception V3 with 299x299 inputs (**A-D**), multi-output Inception V3 with 764x764 inputs (**D-G**), and patch-wise high resolution model (**H-K**). 95% Confidence Intervals (CI), generated using 2,000 bootstrap samples, are visualised as pale curves.

**
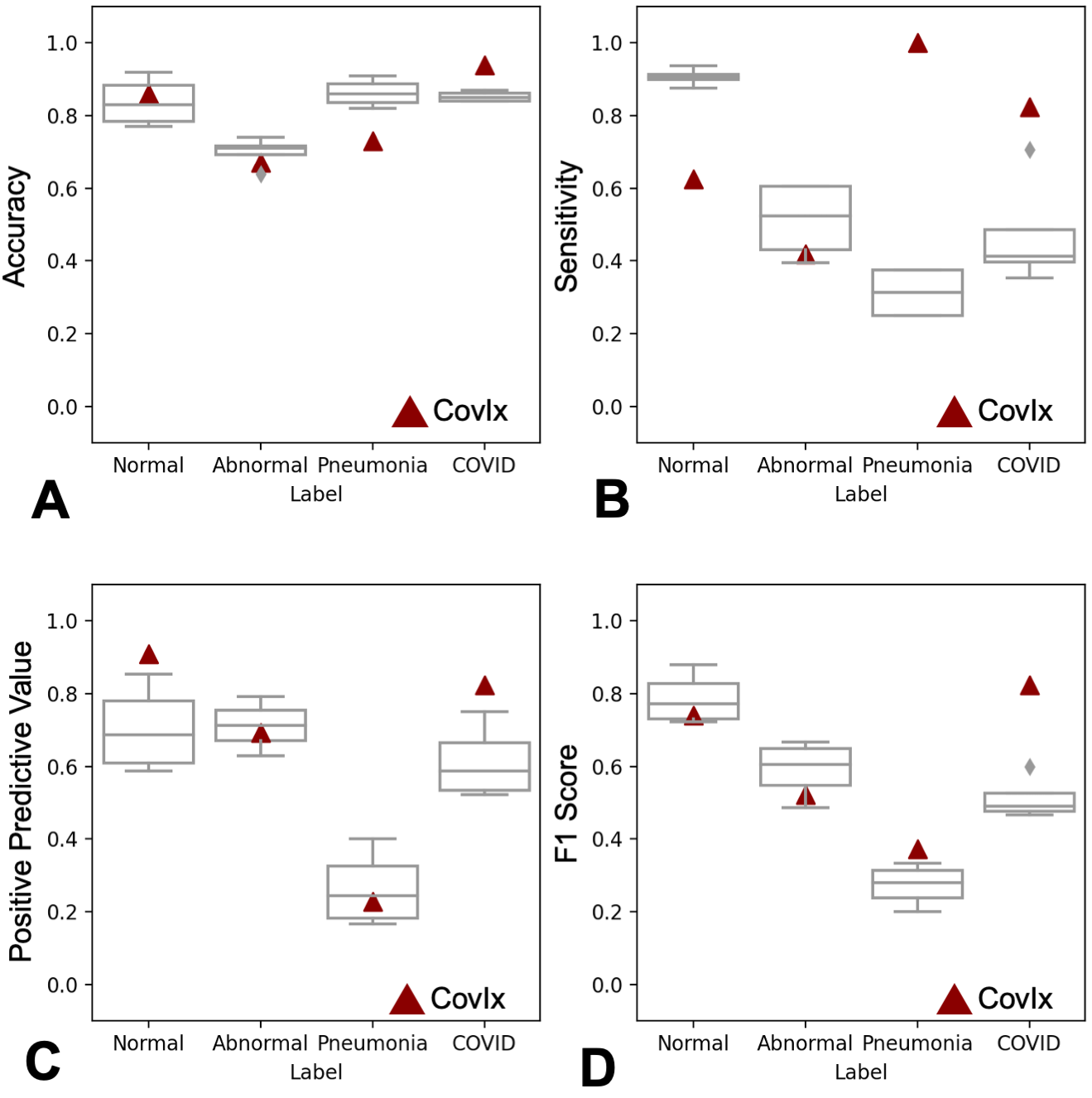
**

**Figure S2. CovIx performance comparison with board-certified radiologists.** Box and whisker plots denote performance distribution of board-certified radiologists, whilst red triangles reflect CovIx performance. The box shows the quartiles of the dataset while the whiskers extend to show the rest of the distribution, except for points that are determined to be “outliers” using a method that is a function of the inter-quartile range. Outliers are shown as grey diamonds.
